# *In vitro* interaction of naphthoquine with ivermectin, atovaquone, curcumin, and ketotifen in the asexual blood stage of *Plasmodium falciparum* 3D7

**DOI:** 10.1128/spectrum.00630-24

**Published:** 2024-05-23

**Authors:** Ruotong Liu, Guoming Li, Mei Li, Baogang Wang, Dongna Zhang, Likun Xu, Liangliang Zhao, Ruhe Liao, Qin Xu, Zhu-Chun Bei, Yabin Song

**Affiliations:** 1 Artemisinin Research Center, Guangzhou University of Chinese Medicine, Guangzhou, China; 2 State Key Laboratory of Pathogen and Biosecurity, Beijing Institute of Microbiology and Epidemiology, Beijing, China; Institut de Recherche pour le Développement, Montpellier, France

**Keywords:** antimalarial drugs, *Plasmodium falciparum*, malaria, pharmacodynamics, drug interactions

## Abstract

Naphthoquine is a promising candidate for antimalarial combination therapy. Its combination with artemisinin has demonstrated excellent efficacy in clinical trials conducted across various malaria-endemic areas. A co-formulated combination of naphthoquine and azithromycin has also shown high clinical efficacy for malaria prophylaxis in Southeast Asia. Developing new combination therapies using naphthoquine will provide additional arsenal responses to the growing threat of artemisinin resistance. Furthermore, due to its long half-life, the possible interaction of naphthoquine with other drugs also needs attention. However, studies on its pharmacodynamic interactions with other drugs are still limited. In this study, the *in vitro* interactions of naphthoquine with ivermectin, atovaquone, curcumin, and ketotifen were evaluated in the asexual stage of *Plasmodium falciparum* 3D7. By using the combination index analysis and the SYBR Green I-based fluorescence assay, different interaction patterns of selected drugs with naphthoquine were revealed. Curcumin showed a slight but significant synergistic interaction with naphthoquine at lower effect levels, and no antagonism was observed across the full range of effect levels for all tested ratios. Atovaquone showed a potency decline when combined with naphthoquine. For ivermectin, a significant antagonism with naphthoquine was observed at a broad range of effect levels below 75% inhibition, although no significant interaction was observed at higher effect levels. Ketotifen interacted with naphthoquine similar to ivermectin, but significant antagonism was observed for only one tested ratio. These findings should be helpful to the development of new naphthoquine-based combination therapy and the clinically reasonable application of naphthoquine-containing therapies.

**IMPORTANCE:**

Pharmacodynamic interaction between antimalarials is not only crucial for the development of new antimalarial combination therapies but also important for the appropriate clinical use of antimalarials. The significant synergism between curcumin and naphthoquine observed in this study suggests the potential value for further development of new antimalarial combination therapy. The finding of a decline in atovaquone potency in the presence of naphthoquine alerts to a possible risk of treatment or prophylaxis failure for atovaquone–proguanil following naphthoquine-containing therapies. The observation of antagonism between naphthoquine and ivermectin raised a need for concern about the applicability of naphthoquine-containing therapy in malaria-endemic areas with ivermectin mass drug administration deployed. Considering the role of atovaquone–proguanil as a major alternative when first-line artemisinin-based combination therapy is ineffective and the wide implementation of ivermectin mass drug administration in malaria-endemic countries, the above findings will be important for the appropriate clinical application of antimalarials involving naphthoquine-containing therapies.

## INTRODUCTION

Malaria remains a major endemic disease threatening human health, causing an estimated 249 million malaria cases and 608,000 deaths worldwide in 2022 (1).The adoption of artemisinin-based combination therapies (ACTs) as first-line treatments for uncomplicated malaria has greatly contributed to the success in reducing the global malaria burden over the last 15 years. However, the emergence and spread of artemisinin partial resistance and subsequent partner drug resistance have resulted in significant failure rates of ACTs, posing a serious threat to global malaria control and elimination efforts (1, 2). Protecting the efficacy of ACTs and developing new non-artemisinin-based combinations (non-ACT) as a contingency against the emergence of artemisinin resistance have been a top priority for the global malaria community (3, 4).

In addition to developing next-generation antimalarial treatments based on innovative molecules, innovation with existing antimalarials, such as triple-ACT (5), or recombination of available antimalarials, is another potential solution. Naphthoquine (NQ) is a 4-aminoquinoline antimalarial first synthesized in China (6) and subsequently developed as a single-dose, fixed co-formulation with artemisinin for uncomplicated malaria treatment (7). The artemisinin–NQ combination, under the name ARCO, is now widely available in malaria-endemic countries and has demonstrated high clinical cure rates for *Plasmodium falciparum* and *Plasmodium vivax* malaria (7–11). Given its long elimination half-life of up to 23 days in humans, NQ has also been considered a potential candidate for the development of single-dose treatment or long-term chemoprophylaxis of malaria (9, 12, 13). Recently, the co-formulated combination of NQ and azithromycin has shown a good safety profile and >90% protective efficacy against malaria with a monthly single-dose regimen in the Greater Mekong Subregion (14, 15). Given the excellent efficacy and good safety profile of NQ-containing therapies shown in clinical trials conducted across different endemic areas, NQ should be a promising candidate for the development of non-ACT or triple-ACT therapies. Potential synergism between NQ and other drugs is worth exploring. In addition, according to its long half-life, there also needs to be concern about a possible interaction between NQ and other drugs, such as drugs used for mass drug administration (MDA) in malaria-endemic areas or antimalarials as an alternative following the failure of ACT treatment. However, the study of the pharmacodynamic interaction between NQ and other drugs is still limited.

This study aimed to explore the potential benefits or limitations of combining NQ with some other drugs for asexual stage parasite inhibition. The *in vitro* drug interactions between NQ and selected partner drugs were investigated in *P. falciparum* 3D7. Using the SYBR Green I-based fluorescence assay for drug inhibition assessment, combination indices derived from combinations with fixed concentration ratios are used to determine the antagonistic or synergistic interaction. Partner drugs selected included ivermectin (IVM), which has been used for MDA or triple-ACT to control malaria transmission and was considered a promising new therapy for fighting malaria (16, 17), and atovaquone (ATO), which is used in fixed combination with proguanil (ATO–proguanil, Malarone) as an alternative when ACT treatment is ineffective and as an option combined with artemisinins in malaria-endemic areas (18). In addition, curcumin (CUR), which demonstrated antimalarial effects through various mechanisms (19, 20), and ketotifen (KTO), which exhibited a highly potent ability to block oocyst development in addition to some activity against asexual stages and gametocytes (21, 22), were also included.

## RESULTS

**Fig 1:**
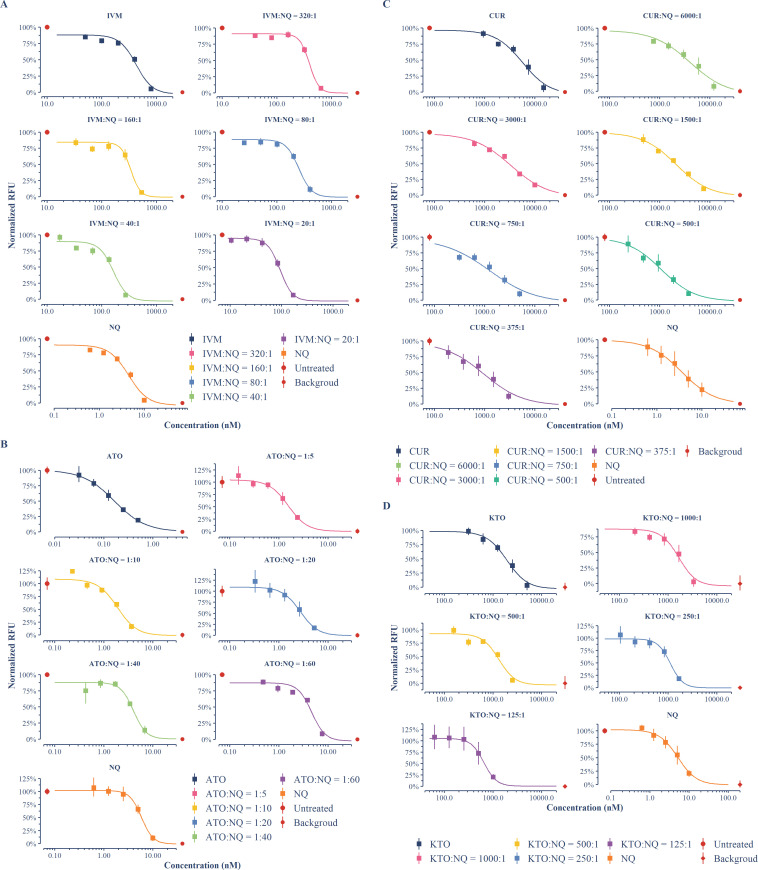
Concentration-dependent inhibition of asexual-stage *P. falciparum* by the combinations of NQ and other drugs at fixed molar ratios. Each data point represents the mean value derived from two independent experiments with triple samples in each, and the error bars depict the 95% CI. Concentration–response curves for combinations of NQ with IVM (**A**), ATO (**B**), CUR (**C**), or KTO (**D**) at various fixed molar ratios were fitted using the four-parameter log-logistic model. Concentration–response curves for individual drugs parallel tested with combinations were also provided.

IVM, ATO, CUR, and KTO were chosen as partner drugs to be combined with NQ in this study. To probe the types of their interactions with NQ, each of them was combined with NQ at various molar ratios and tested on the asexual stage of *P. falciparum* 3D7, with individual drugs tested in parallel. Using the 72-h SYBR Green I-based fluorescence assay, all individual drugs and fixed-ratio combinations showed a good concentration-dependent manner for inhibition (Fig. 1; Table 1). The NQ presented half maximal inhibitory concentrations (IC_50_s) ranging from 3.60 to 5.84 nM, which aligns with previous findings (23, 24). The other individual drugs tested also exhibited potency similar to those reported in other studies (16, 21, 25–27). ATO demonstrated the highest potency, with an IC_50_ nearly 40 times lower than that of NQ, reaching the sub-nanomolar range. CUR and KOT, on the other hand, showed apparently weak inhibition on asexual-stage parasites, with IC_50_s up to micromolar levels. IVM exhibited moderate activity, with an IC_50_ approximately 100 times higher than that of NQ. Combinations with various ratios surrounding the IC_50_ ratios of partner drugs to NQ showed potency between that of NQ and partner drugs combined.

**TABLE 1:** *In vitro* efficacy of NQ-containing combinations and individual drugs for combination on the asexual stage of *P. falciparum 3D7*

Drug combination	Combination ratio^*a*^	IC_50_ (nM)^*b*^		
		Combination^*c*^	Individual drug^*d*^	
IVM + NQ (*n* = 3)			IVM (*n* = 6)	NQ (*n* = 6)
	320:1	392.74 [362.73, 422.75]	424.59 [391.84, 457.33]	4.46 [3.96, 4.96]
	160:1	338.98 [296.62, 381.34]		
	80:1	252.55 [230.16, 274.95]		
	40:1	162.95 [146.24, 179.66]		
	20:1	93.21 [86.56, 99.86]		
ATO + NQ (*n* = 2)			ATO (*n* = 4)	NQ (*n* = 4)
	1:60	4.61 [4.03, 5.19]	0.16 [0.13, 0.20]	5.84 [5.00, 6.68]
	1:40	4.01 [3.28, 4.74]		
	1:20	2.72 [1.88, 3.56]		
	1:10	1.89 [1.56, 2.23]		
	1:5	1.55 [1.19, 1.90]		
CUR + NQ (*n* = 2)			CUR (*n* = 4)	NQ (*n* = 4)
	6000:1	4,051.08 [2,781.55, 5,320.61]	5,698.11 [4,601.23, 6,794.98]	3.60 [2.07, 5.13]
	3000:1	3,308.28 [2,787.69, 3,828.86]		
	1500:1	2,128.35 [1,775.87, 2,480.83]		
	750:1	1,261.12 [855.08, 1,667.16]		
	500:1	1,072.45 [709.11, 1,435.78]		
	375:1	960.21 [525.96, 1,394.46]		
KTO + NQ (*n* = 2)			KTO (*n* = 4)	NQ (*n* = 4)
	1000:1	1,654.36 [1,202.90, 2,105.83]	1,921.99 [1,575.02, 2,268.96]	5.13 [3.91, 6.35]
	500:1	1,313.67 [1,056.78, 1,570.55]		
	250:1	1,108.96 [908.76, 1,309.15]		
	125:1	644.74 [431.32, 858.16]		

^*a*^ The molar ratio of partner drug to NQ.

^*b*^ IC_50_s given as the estimate (lower, upper 95% confidence limit] were estimated by the concentration–response curves fitted using data from at least two independent experiments with triplet samples.

^*c*^ Sum of the individual drug concentrations.

^*d*^ IC_50_s for each individual drug assayed in parallel with combinations.

**Fig 2:**
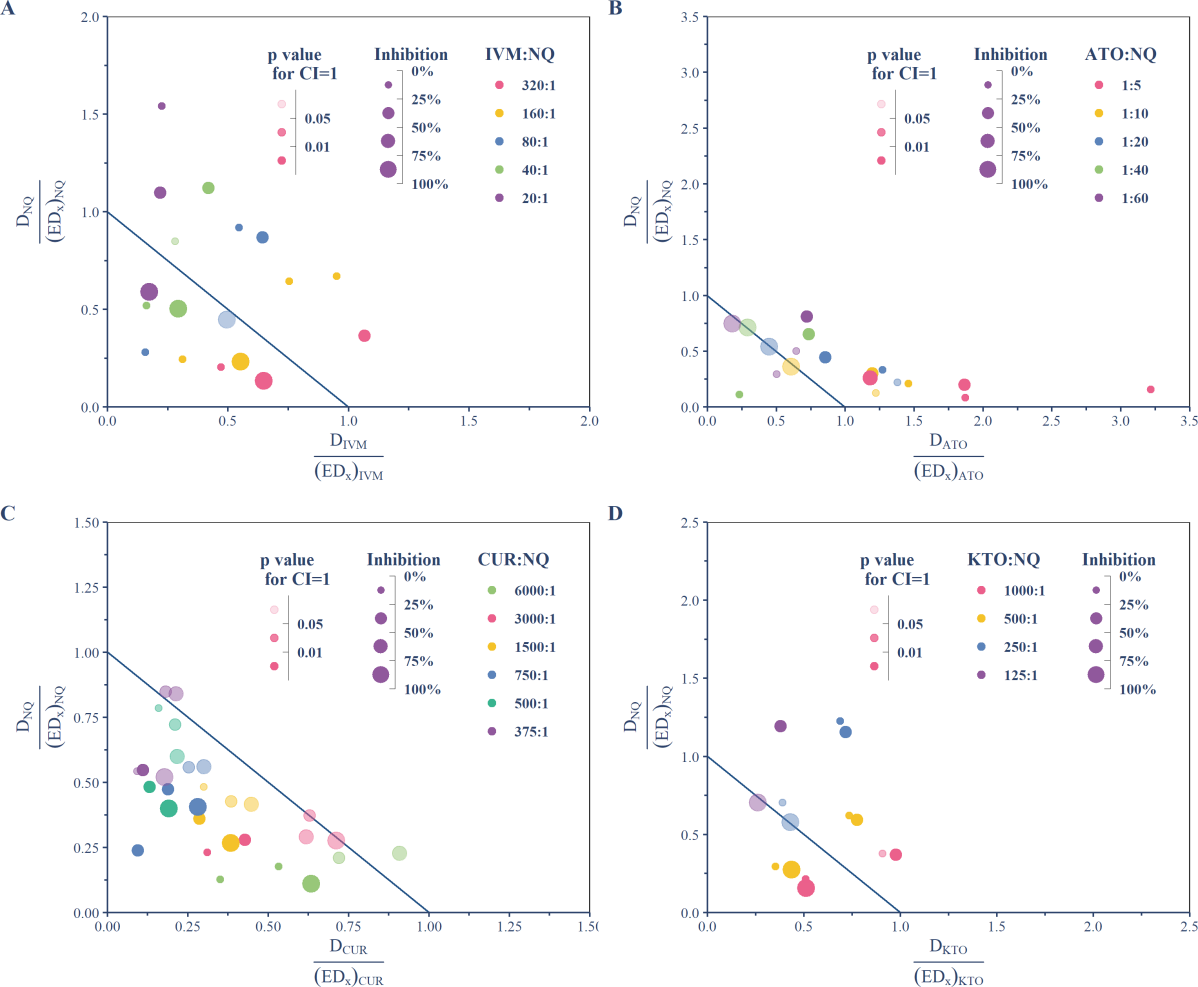
Normalized isobolograms of the combinations of NQ and other drugs against *P. falciparum* 3D7. Normalized isobolograms were created using data points with inhibition levels above 5%. The *D*_partner_drug_, *D*_NQ_, (ED_*x*_)_partner_drug_, and (ED_*x*_)_NQ_ are the concentrations of partner drug and NQ in combination or individually to produce inhibition *x*. The colors and sizes of circles indicate different ratios for combination and inhibition levels achieved; the alpha transparency scales in circle color reflect the *P* value of *z*-testing for CI = 1. Data points on, below, or above the blue line indicate additive interaction, synergism, or antagonism, respectively. (**A**) Combinations of IVM and NQ; (**B**) combinations of ATO and NQ; (**C**) combinations of CUR and NQ; (**D**) combinations of KTO and NQ.

**Fig 3:**
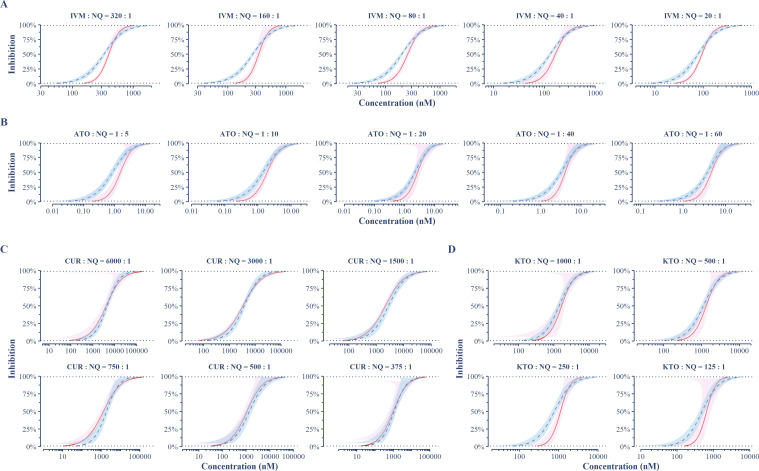
Comparison between the concentration–inhibition curves derived from the fitted model and those from the additive effect assumption. (**A**) Combinations of IVM and NQ; (**B**) combinations of ATO and NQ; (**C**) combinations of CUR and NQ; (**D**) combinations of KTO and NQ; the blue dashed line is the reference line under the additive effect assumption, and the red solid line is the fitted curves of concentration–inhibition for the combinations. Left and right shifts of the fitted line (red) relative to the reference line (blue) indicate synergism and antagonism, respectively. The shaded areas indicate the 95% CI.

To determine the types of interaction in each combination, data points with significant inhibition (>5%) were used for CI determination (Table S1) and the normalized isobologram (Fig. 2). CUR combined with NQ showed synergistic to additive interactions, with nearly all data points falling in the lower left part of the normalized isobologram and having CI values ranging from 0.33 to 1.14 (mean = 0.77 [0.71, 0.83]) (Fig. 2C). ATO acted with NQ mainly in additive or antagonistic manners, with CI values ranging from 0.35 to 3.37 (mean = 1.42 [1.169, 1.71]), and having only one data point (~15% inhibition) showed significant synergism (Fig. 2B; Table S1). IVM or KTO combined with NQ showed interaction types ranging from antagonism to synergism, according to the combination ratios and inhibition levels achieved, with CI values ranging from 0.44 to 1.77 (mean = 1.09 [0.90, 1.27]) and from 0.67 to 1.91 (mean = 1.18 [0.98, 1.41])] respectively (Fig. 2A and D). Based on the fitted concentration–response curves, the CI values at IC_50_ and IC_90_ for each combination were also determined (Table 2), showing significant antagonism at IC_50_ but a more likely additive effect at IC_90_ for IVM and ATO, interactions from significant synergistic to additive at IC_50_ but only additive at IC_90_ for CUR, and an additive effect for KTO combined with NQ at both IC_50_ and IC_90_, except for one ratio of 250:1 showing significant antagonism at CI_50_. To further illuminate the diverse types of interaction at various combination ratios and inhibition levels, the concentrations of each combination required for inhibition levels from 1% to 99% were predicted under assuming additive interaction, which then was compared to the estimated values derived from concentration–response curves fitted using actual data. Except for CUR, other drugs combined with NQ resulted in a right shift in the concentration–inhibition curves relative to the additive reference curve at lower inhibition levels (Fig. 3). It indicates an antagonistic interaction between them and NQ at the lower inhibition range, notably IVM, ATO with combination ratios above 1:10, and KTO with combination ratios of 250:1, which have a significant right shift at a broad inhibition range below ~75%. In contrast, CUR combined with NQ resulted in a left shift in the lower inhibition range of concentration–response curves (Fig. 3). Despite the slight left shift, in the combinations with ratios of 1,500:1 and 750:1, it is significant (*P* < 0.05, *z*-test for CI = 1), indicating a significant synergism.

**TABLE 2:** The *in vitro* interactions of NQ with IVM, ATO, CUR, and KTO against the asexual stage of *P. falciparum 3D7*

Drug combination	Combination ratio	CI values^*a*^ for	
		IC_50_	IC_90_
IVM + NQ	320:1	1.20 [1.07, 1.31]**	0.81 [0.62, 1.01]
	160:1	1.27 [1.09, 1.44]**	0.83 [0.57, 1.09]
	80:1	1.29 [1.14, 1.43]***	0.93 [0.70, 1.57]
	40:1	1.27 [1.10, 1.43]**	0.91 [0.61, 1.21]
	20:1	1.20 [1.06, 1.34]	0.80 [0.60, 1.00]
ATO + NQ	1:60	1.24 [1.03, 1.45]*	1.04 [0.57,1.51]
	1:40	1.27 [0.99, 155]	0.94 [0.47, 1.41]
	1:20	1.24 [0.82, 1.65]	1.00 [0.33, 1.67]
	1:10	1.35 [1.02, 1.68]*	1.01 [0.56, 1.46]
	1:5	1.80 [1.27, 2.33]**	1.15 [0.47, 1.84]
CUR + NQ	6000:1	0.90 [0.58, 1.21]	1.33 [0.33, 2.33]
	3000:1	0.89 [0.67, 1.10]	1.26 [0.64, 1.89]
	1500:1	0.77 [0.55, 0.98]*	0.86 [0.42, 1.30]
	750:1	0.69 [0.39, 0.98]*	1.30 [0.21, 2.40]
	500:1	0.78 [0.42, 1.14]	0.78 [0.13, 1.43]
	375:1	0.88 [0.39, 1.36]	1.13 [0.06, 2.32]
KTO + NQ	1000:1	1.18 [0.83, 1.54]	0.97 [0.37, 1.58]
	500:1	1.19 [0.91, 1.48]	0.91 [0.45, 1.37]
	250:1	1.44 [1.09, 1.77]*	0.88 [0.45, 1.30]
	125:1	1.33 [0.84, 1.82]	0.80 [0.20, 1.40]

^*a*^ CI values shown as estimate [lower, upper 95% confidence limit] were determined using IC_50_s and IC_90_s derived from the fitted concentration–response curves of each combination and individual drug. The one-sample *z*-test was used for testing CI = 1. The * , ** , and *** indicate *P* values of <0.05, <0.01, and <0.001, respectively.

## DISCUSSION

Pharmacodynamic interaction study is important for combination therapy because the antagonistic interaction reducing drug efficacy in combination could result in inadequate treatment, which increases the risk of treatment failure and *de novo* selection of resistant parasites (28). Previous *in vitro* studies on *P. falciparum* using isobologram analysis showed that NQ has no significant interaction with piperaquine, mefloquine, or quinine, but is weakly antagonistic with dihydroartemisinin and chloroquine (29), and synergistic with primaquine (23) and tafenoquine (24). In this study, pharmacodynamic interactions between NQ and IVM, ATO, CUR, or KTO were investigated on asexual-stage *P. falciparum* 3D7. Unlike previous studies that often evaluated the interactions at a single effect level of IC_50_, we assessed the interactions at various effect levels for each combination. CUR appeared somewhat promising, showing no significant antagonistic interaction with NQ at all tested ratios but significant synergism with NQ at some ratios (Fig. 2C and 3C; Table 2). The other three seem to interact with NQ in a similar pattern, which is additive to slightly synergistic at higher inhibition levels but antagonistic at lower inhibition levels (Fig. 2 and 3).

CUR, or diferuloylmethane, is a polyphenol isolated from turmeric (the roots of *Curcuma longa*), which has broad biological and pharmacological activities, including antioxidant, anti-inflammatory, anticarcinogenic, antimicrobe, and so on. It seems to exert antimalarial effects through various mechanisms (19, 20). Previous research by Cui et al. (30) revealed that CUR increased intracellular reactive oxygen species (ROS) levels, leading to cytotoxicity on *P. falciparum*, which can be counteracted by antioxidants or ROS scavengers. This suggests that ROS is a major effector of CUR in killing malaria parasites. In *Plasmodium*, the accumulation of free heme released *via* hemoglobin proteolysis also generates ROS, which induces oxidative stress leading to parasite death (31). Although the action mechanism of NQ remains to be elucidated, it is thought to be similar to that of other 4-aminoquinolines; binding with free heme to impede hemozoin biocrystallization, interfering with heme detoxification primarily contributes to its schizonticide activity (11) . Hence, the observed synergism between CUR and NQ in this study may be explained by a cooperative effect of CUR-induced ROS increase and NQ’s inhibition on heme detoxification in counteraction to the parasite’s antioxidant defense system. Both our results and previous studies (26, 27) showed that the *in vitro* antimalarial activity of CUR is not so strong. However, a previous study illustrated that nanonization of CUR could enhance its *in vitro* activity against *P. falciparum* by 10-fold (27). Recent exploration of combining CUR with other antimalarial drugs in nanoformulations have also presented encouraging results in the rodent malaria model (32–34). It is worthy to be noticed that, besides its direct cytotoxic effect on parasites, the immunomodulatory and anti-inflammatory effects of CUR have also been demonstrated to enhance parasite-infected erythrocyte phagocytosis, suppress inflammation, protect against endothelial brain damage caused by parasite-infected erythrocyte sequestration, and prevent parasite relapse (19, 20). Combined with all of these, the finding of no significant antagonism but synergism between CUR and NQ in this study suggests a potential value of their combination (maybe as a nanoformulation) for developing novel antimalarial combination therapy.

The underlying mechanism for the interactions between NQ and other tested drugs is unclear. The normalized isobologram showed that the combination data points of ATO and NQ are biased to fall in the lower right region, especially with the ratio of 1:5 (Fig. 2B). This indicates that the antagonism between ATO and NQ is mainly due to a decline in ATO potency, especially as the proportion of ATO increases. ATO is a mitochondrial electron transport chain inhibitor targeting cytochrome *b*, which is active against both liver- and blood-stage malaria parasites (25). ATO is used as a fixed-dose combination with proguanil, which is commonly used for malaria prophylaxis or uncomplicated malaria treatment in travelers and serves as an alternative treatment when first-line ACT is unavailable or ineffective (18). It was also considered a potentially attractive option for triple-ACT (5). Given the long-elimination half-life of NQ, the above finding suggests a possible decrease in ATO–proguanil efficacy following NQ-containing therapy (e.g., artemisinin–NQ for treatment, NQ–azithromycin for prophylaxis), alerting a potential risk of treatment failure.

IVM is a broad-spectrum antiparasitic drug with endectocide activity. It primarily targets the glutamate-gated chloride (GluCl) ion channels in postsynaptic neurons and neuromuscular junctions of invertebrates. Blocking the closure of the GluCl channel causes the hyperpolarization of neurons and muscle fibers, leading to subsequent flaccid paralysis or death of the insect (17). The MDA of IVM has been widely used to eradicate onchocerciasis, and lymphatic filariasis, and treat several other parasitic diseases in humans. In *Plasmodium*, IVM has shown multi-stage inhibitory activity, including inhibition on the development of blood asexual and sexual stages of *P. falciparum* (35), liver stages of *P. berhgei* (36), and liver schizonts and hypnozoites of *P. cynomolgi* (37). Recently, IVM has been proposed as a complementary malaria vector control tool based on its significant mosquito-lethal effect on many species of *Anopheline* mosquitoes and sporontocidal effect on malaria parasites (38). IVM alone or combined with ACTs in MDA is under investigation for reducing malaria transmission, and several field trials have shown promising results in reducing wild *Anopheles* survival and human malaria incidence (16, 17, 39). Although the main target of IVM MDA is mosquitoes feeding on humans, the possible pharmacodynamic interaction, especially the antagonistic interaction between IVM and other antimalarials on asexual blood stage parasites, should also be cause for concern. Our finding on the interaction between IVM and NQ suggests that both the application of NQ-containing therapies in endemic areas with IVM MDA deployed or the deployment of MDA of IVM combined with NQ-containing therapies may need to be prudent. Although IVM seems not to interact antagonistically with NQ at high effect levels (>75%), the possible effect of significant antagonism observed at lower but broad-range effect levels (Table 2; Fig. 3A) needs to be further investigated.

KTO was included in this study due to its high sporontocidal and gametocidal activity in malaria parasites (22) both of which may provide additional transmission-blocking benefits. The result showed that KTO interacted with NQ in a manner similar to IVM, but with less significance for the antagonism at lower effect levels, especially when it combined with NQ at higher ratios (Table 2; Fig. 3D). Considering the higher peak plasma drug concentrations (1.92 µg/mL after multiple oral doses of 1 mg twice daily in adult humans) (22) compared to IVM (105.2  ng/mL after 3-day doses of 600 µg/kg daily) (16) that can be achieved in clinical practice, KTO may be more suitable to be combined with NQ for additional transmission-blocking benefits.

In conclusion, CUR showed slight but significant synergism with NQ, suggesting a potential value for its combination with NQ; ATO suffered potency decline in combination with NQ, alerting a possible failure risk of ATO–proguanil treatment after NQ-containing therapies. Additionally, antagonistic interaction at lower effect levels was observed for both IVM and KTO combined with NQ, but it is less significant for the latter. These findings should be helpful to the development of new NQ-based combination therapies and the clinically reasonable application of NQ-containing therapies.

## MATERIALS AND METHODS

### Drugs and chemical reagents

NQ phosphate was purchased from Shanghai New Hualian Pharmaceutical Co., Ltd. (Shanghai, China). ATO (Product no.: PHR1591), IVM (Product no.: PHR1380), and CUR (Product no.: 78246) were purchased from Sigma-Aldrich. KTO fumarate was purchased from MedChemExpress (Shanghai, China). NQ was dissolved in saline solution at 5 mg/mL, and CUR, KTO, ATO, and IVM were dissolved in dimethylsulfoxide (DMSO) (Innochem, Beijing, China) at 10 mg/mL to make a stock solution. Stock solutions were sterilized using a syringe filter with 0.2-µm Supor membrane (Pall Life Sciences, Portsmouth, UK) and then stored at −20°C until ready for use. Working drug solutions were freshly prepared by serial dilution in the parasite culture medium just before the drug inhibition assay setup. RPMI 1640 medium containing L-glutamine and 25 mM of 4-(2-hydroxyethyl)-1-piperazineethanesulfonic acid (HEPES) was purchased from Gibco Life Technologies (Grand Island, NY, USA), and Albumax II was purchased from Gibco Thermo Fisher Scientific (Penrose, Auckland, New Zealand). Hypoxanthine and SYBR Green I nucleic acid gel stain (10,000× in DMSO) was purchased from Sigma-Aldrich.

### Parasite culture

*P. falciparum* 3D7 strain was maintained in O^+^ human red blood cells (RBCs) using the method of Trager and Jensen with some modifications (40, 41). O^+^ RBCs were from healthy donors. Briefly, asexual-stage parasites were grown in O^+^ human RBC using parasite culture medium, namely, RPMI 1640 medium containing 25 mM of NaHCO_3_, 25 mM of HEPES, and 11 mM of D-glucose and supplemented with 0.5% Albumax II, 50 mg/L of hypoxanthine, 100 U/L of penicillin, and 100 µg/L of streptomycin at 37°C under a 5% O_2_, 5% CO_2_, balanced N_2_ atmosphere. The culture was routinely maintained with daily medium changes and subculture by the addition of fresh RBCs every 4–5 days.

### Parasite growth-inhibition assay

The inhibition of drugs on asexual-stage parasite growth was assessed using the previously described SYBR Green I-based fluorescence assay with minor modification (42). Parasite cultures were synchronized by 5% D-sorbitol (wt/vol) to enrich ring-stage parasite and adjusted to 1% parasitemia and 8% hematocrit by adding fresh RBCs and culture medium. Of the ring-stage enriched cultures, 50 µL was aliquoted into pre-loaded black 96-well flat-bottom plates containing 50 µL of serial dilutions of working drug solutions at 2× test concentrations to make a final volume of 100 µL per well and 1% parasitemia, 4% hematocrit. In each plate, wells without drugs and wells with only RBCs were included for untreated controls and background controls, respectively. The plates were incubated at 37°C under the hypoxic atmosphere described above for 72 h. To measure the parasite growth, 100 µL of lysis buffer (20 mM Tris [pH 7.5], 5 mM EDTA, 0.008% [wt/vol] saponin, and 0.08% [vol/vol] Triton X-100) containing SYBR Green I (1× final concentration) was added directly to each well and mixed gently. After 3 h of incubation in the dark, the SYBR Green I fluorescence corresponding to parasite density was then determined using SpectraMax i3x microplate reader (Molecular Devices, San Jose, CA, USA) set to an excitation wavelength of 490 nm and emission wavelength of 520 nm. All the acquired fluorescence intensity values were normalized by taking the mean value of untreated controls as 100% and that of background controls as 0%. Taking the normalized relative fluorescence unit as response, the concentration–response curves were fitted using the four-parameter log-logistic model. The IC_50_s or other needed inhibitory concentrations were estimated according to the fitted concentration–response curves.

### Drug combination assays

NQ was combined with IVM, ATO, CUR, and KTO at fixed molar ratios (Table 1). Drug combination ratios were chosen according to the IC_50_ of each individual drug to ensure proper concentration–response curve fitting. The fixed-ratio combinations were subjected to parasite growth inhibition assay as a new drug, taking the sum of each individual drug’s concentration as its concentration. For each combination, at least two independent assays were performed, each in triplicate, and individual drugs were set up in parallel in each assay. For drug–drug interaction analysis, the combination index (CI) theorem (43, 44) was used. The fitted concentration–response curves were used to estimate the inhibitory concentrations for CI calculation using equation (1). In which *D_A,x_*, and *D_B,x_* are the concentrations of drug A and drug B in combination to produce effect *x* (e.g., 50% inhibition); *E_x, A_* and *E_x, B_* are the concentrations of drug A and drug B individually to produce the same effect. CI = 1, < 1, and > 1 indicate additive effect, synergism, and antagonism, respectively, resulting in the combination data points falling on the hypothenuse (line with intercept = 1 and slope = −1), on the lower left, and on the upper right of the normalized isobologram created by plotting $\frac{D_{A,x}}{E_{x,A}}$ vs $\frac{D_{B,x}}{E_{x,B}}$ .


(1) $$CI=\frac{D_{A,x}}{E_{x,A}}+\frac{D_{B,x}}{E_{x,B}}$$

The different levels of inhibitory concentrations for combinations under the additive effect (CI = 1) assumption were also calculated to plot the additivity reference curve in the concentration–inhibition plotting, where a left or right shift of the fitted model-predicted curve to reference indicates synergism or antagonism.

### Statistical analysis

Statistical analysis was performed using R v4.3.1 (45) in RStudio (46). The four-parameter log-logistic model-based concentration–response curve fitting, IC_50_ estimation, and CI calculation were performed with the assistance of the R package drc (47). The one-sample *z*-test was used for testing CI = 1, and a *P* value of <0.05 was considered significant. For the graphic presentation of results, the R package ggplot2 (48) was also used.

## Data Availability

The authors confirm that the data supporting the findings of this study are available within the article and its supplemental material.

## SUPPLEMENTAL MATERIAL

Tables S1 spectrum.00630-24-s0001.pdf Source data for Fig. 2.

ASM does not own the copyrights to Supplemental Material that may be linked to, or accessed through, an article. The authors have granted ASM a non-exclusive, world-wide license to publish the Supplemental Material files. Please contact the corresponding author directly for reuse.
