# Supplementary material for: In vitro interaction of naphthoquine with ivermectin, atovaquone, curcumin, and ketotifen in the asexual blood stage of Plasmodium falciparum 3D7
Source: Microbiol Spectr. 2024 May 23;12(7):e00630-24. doi: 10.1128/spectrum.00630-24 (PMC11218538; doi:10.1128/spectrum.00630-24)
Supplement: Tables S1 — Source data for Fig. 2. [file spectrum.00630-24-s0001.pdf]

Table S1：Combination indecs for the fixed-ratio combinations of NQ and chosen partner drugs tested in this study. Data derived from 2 or 3 (the IVM-NQ combinations) independent experiments with triple samples. The *p value* is from the z-test for CI=1.

Drugs		Concentration (nM)				Mean of Normalized RFU	Inhibition	IC_x (nM)				Combination Index (CI)			
								Estimate		Std. Error		Estimate	95% LCI	95% UCI	*p* value
A	B	A	B	A+B	A/B			A	B	A	B				
IVM	NQ	640	2	642	320	0.072278445	91.96%	988.65	14.78	100.93	1.83	0.78	0.65	0.92	0.0014
IVM	NQ	320	1	321	320	0.664617864	26.94%	300.42	2.73	19.67	0.26	1.43	1.28	1.58	0.0000
IVM	NQ	80	0.25	80.25	320	0.849452369	6.65%	169.89	1.22	22.14	0.22	0.68	0.54	0.82	0.0000
IVM	NQ	533.333	3.33333	536.667	160	0.066889468	91.46%	966.00	14.31	96.03	1.72	0.79	0.66	0.91	0.0005
IVM	NQ	266.667	1.66667	268.333	160	0.645941267	23.23%	280.49	2.48	20.39	0.26	1.62	1.43	1.82	0.0000
IVM	NQ	133.333	0.83333	134.167	160	0.779900155	7.44%	177.15	1.29	22.20	0.22	1.40	1.11	1.68	0.0065
IVM	NQ	66.6667	0.41667	67.0833	160	0.739577773	12.19%	214.11	1.69	22.06	0.24	0.56	0.46	0.65	0.0000
IVM	NQ	400	5	405	80	0.111901726	86.55%	809.73	11.14	64.33	1.06	0.94	0.83	1.06	0.3241
IVM	NQ	200	2.5	202.5	80	0.623853435	29.03%	311.42	2.88	19.27	0.26	1.51	1.34	1.68	0.0000
IVM	NQ	100	1.25	101.25	80	0.809565785	8.17%	183.45	1.36	22.23	0.23	1.47	1.14	1.79	0.0056
IVM	NQ	25	0.3125	25.3125	80	0.832458432	5.59%	159.36	1.11	21.99	0.21	0.44	0.33	0.55	0.0000
IVM	NQ	266.667	6.66667	273.333	40	0.064975565	90.07%	912.25	13.19	84.68	1.47	0.80	0.67	0.92	0.0013
IVM	NQ	133.333	3.33333	136.667	40	0.616346555	30.41%	318.62	2.97	19.00	0.26	1.54	1.34	1.74	0.0000
IVM	NQ	66.6667	1.66667	68.3333	40	0.751088762	15.83%	237.83	1.96	21.63	0.25	1.13	0.91	1.35	0.2472
IVM	NQ	33.3333	0.83333	34.1667	40	0.795442911	11.03%	205.82	1.60	22.15	0.24	0.68	0.53	0.84	0.0001
IVM	NQ	160	8	168	20	0.082308513	90.55%	929.73	13.55	88.33	1.55	0.76	0.63	0.90	0.0006
IVM	NQ	80	4	84	20	0.56738568	39.78%	367.73	3.64	17.31	0.25	1.32	1.16	1.47	0.0000
IVM	NQ	40	2	42	20	0.875930833	7.49%	177.54	1.30	22.21	0.22	1.77	1.24	2.29	0.0042
ATO	NQ	0.4	2	2.4	1/5	0.283017216	72.92%	0.34	7.67	0.05	0.84	1.44	1.11	1.78	0.0096
ATO	NQ	0.2	1	1.2	1/5	0.667901965	36.15%	0.11	4.99	0.01	0.37	2.06	1.61	2.52	0.0000
ATO	NQ	0.1	0.5	0.6	1/5	0.946618057	9.52%	0.03	3.14	0.01	0.47	3.37	1.93	4.82	0.0013
ATO	NQ	0.05	0.25	0.3	1/5	0.963615946	7.90%	0.03	2.97	0.01	0.48	1.95	1.05	2.85	0.0378
ATO	NQ	0.33333	3.33333	3.66667	1/10	0.164745644	83.90%	0.55	9.19	0.10	1.35	0.97	0.73	1.21	0.7989
ATO	NQ	0.16667	1.66667	1.83333	1/10	0.596429182	44.64%	0.14	5.50	0.02	0.39	1.50	1.23	1.77	0.0003
ATO	NQ	0.08333	0.83333	0.91667	1/10	0.874061408	19.39%	0.06	3.95	0.01	0.41	1.67	1.18	2.16	0.0071
ATO	NQ	0.04167	0.41667	0.45833	1/10	0.970114433	10.65%	0.03	3.25	0.01	0.47	1.35	0.82	1.88	0.1939
ATO	NQ	0.25	5	5.25	1/20	0.168034149	84.18%	0.56	9.24	0.10	1.36	0.99	0.76	1.21	0.9184
ATO	NQ	0.125	2.5	2.625	1/20	0.584437974	46.28%	0.15	5.60	0.02	0.40	1.30	1.10	1.50	0.0033
ATO	NQ	0.0625	1.25	1.3125	1/20	0.912332785	16.43%	0.05	3.73	0.01	0.43	1.60	1.14	2.07	0.0105
ATO	NQ	0.03125	0.625	0.65625	1/20	1.022338315	6.41%	0.02	2.80	0.01	0.49	1.60	0.89	2.32	0.0991
ATO	NQ	0.16667	6.66667	6.83333	1/40	0.138629035	84.64%	0.57	9.33	0.11	1.40	1.00	0.77	1.24	0.9678
ATO	NQ	0.08333	3.33333	3.41667	1/40	0.547833954	37.92%	0.11	5.10	0.01	0.37	1.39	1.19	1.59	0.0001
ATO	NQ	0.01042	0.41667	0.42708	1/40	0.750162457	14.82%	0.05	3.61	0.01	0.44	0.35	0.26	0.44	0.0000
ATO	NQ	0.125	7.5	7.625	1/60	0.086484352	87.71%	0.69	10.02	0.14	1.66	0.93	0.68	1.18	0.5805
ATO	NQ	0.0625	3.75	3.8125	1/60	0.606155834	29.79%	0.09	4.61	0.01	0.37	1.53	1.30	1.76	0.0000
ATO	NQ	0.03125	1.875	1.90625	1/60	0.728487004	16.16%	0.05	3.71	0.01	0.43	1.15	0.89	1.41	0.2632
ATO	NQ	0.01563	0.9375	0.95313	1/60	0.787828554	9.54%	0.03	3.15	0.01	0.47	0.80	0.56	1.04	0.1038
CUR	NQ	12000	2	12002	6000	0.077292338	87.34%	18944.67	17.85	3391.42	6.88	0.75	0.51	0.98	0.0359
CUR	NQ	6000	1	6001	6000	0.397633535	55.90%	6603.20	4.38	625.49	0.94	1.14	0.94	1.33	0.1668
CUR	NQ	3000	0.5	3000.5	6000	0.583238068	37.68%	4166.55	2.37	477.07	0.56	0.93	0.74	1.12	0.4721
CUR	NQ	1500	0.25	1500.25	6000	0.718505379	24.40%	2819.86	1.41	431.27	0.43	0.71	0.52	0.90	0.0028
CUR	NQ	750	0.125	750.125	6000	0.791908433	17.20%	2143.41	0.98	397.14	0.35	0.48	0.32	0.63	0.0000
CUR	NQ	10000	3.33333	10003.3	3000	0.161367717	81.05%	14071.13	12.01	2036.61	3.83	0.99	0.72	1.25	0.9308
CUR	NQ	5000	1.66667	5001.67	3000	0.336948904	63.65%	8075.11	5.73	802.64	1.30	0.91	0.73	1.09	0.3194
CUR	NQ	2500	0.83333	2500.83	3000	0.616606639	35.95%	3978.53	2.23	470.78	0.54	1.00	0.77	1.23	0.9877
CUR	NQ	1250	0.41667	1250.42	3000	0.721658083	25.55%	2929.05	1.48	435.61	0.44	0.71	0.50	0.91	0.0050
CUR	NQ	625	0.20833	625.208	3000	0.819828878	15.82%	2014.32	0.90	388.69	0.34	0.54	0.33	0.75	0.0000
CUR	NQ	7500	5	7505	1500	0.101747462	87.91%	19583.35	18.66	3584.77	7.34	0.65	0.40	0.90	0.0058
CUR	NQ	3750	2.5	3752.5	1500	0.335271291	64.95%	8364.52	6.01	845.11	1.39	0.86	0.66	1.07	0.2030
CUR	NQ	1875	1.25	1876.25	1500	0.550610867	43.78%	4877.07	2.93	505.02	0.65	0.81	0.61	1.01	0.0654
CUR	NQ	937.5	0.625	938.125	1500	0.699140095	29.18%	3281.79	1.73	448.27	0.47	0.65	0.44	0.86	0.0009
CUR	NQ	468.75	0.3125	469.063	1500	0.882165217	11.18%	1569.56	0.65	352.65	0.28	0.78	0.35	1.21	0.3238
CUR	NQ	5000	6.66667	5006.67	750	0.099389368	86.16%	17775.27	16.40	3046.05	6.08	0.69	0.38	1.00	0.0486
CUR	NQ	2500	3.33333	2503.33	750	0.317578392	64.72%	8310.68	5.95	837.04	1.37	0.86	0.60	1.12	0.2931
CUR	NQ	1250	1.66667	1251.67	750	0.525125389	44.32%	4943.90	2.98	508.14	0.65	0.81	0.57	1.06	0.1344
CUR	NQ	625	0.83333	625.833	750	0.674762189	29.61%	3325.08	1.76	449.72	0.48	0.66	0.41	0.92	0.0099
CUR	NQ	312.5	0.41667	312.917	750	0.677069355	29.39%	3302.60	1.74	448.97	0.47	0.33	0.20	0.46	0.0000
CUR	NQ	3750	7.5	3757.5	500	0.102271747	87.95%	19629.14	18.71	3598.76	7.37	0.59	0.27	0.91	0.0116
CUR	NQ	1875	3.75	1878.75	500	0.323917145	66.09%	8631.13	6.26	886.26	1.47	0.82	0.54	1.10	0.1965
CUR	NQ	937.5	1.875	939.375	500	0.585893785	40.25%	4456.77	2.60	487.51	0.59	0.93	0.61	1.26	0.6866
CUR	NQ	468.75	0.9375	469.688	500	0.667874523	32.17%	3582.07	1.94	458.07	0.50	0.61	0.37	0.86	0.0022
CUR	NQ	234.375	0.46875	234.844	500	0.89005461	10.25%	1477.62	0.60	343.58	0.27	0.94	0.25	1.64	0.8764
CUR	NQ	3000	8	3008	375	0.122567729	85.17%	16910.55	15.34	2798.31	5.51	0.70	0.33	1.07	0.1122
CUR	NQ	1500	4	1504	375	0.392524881	58.34%	7026.14	4.76	669.39	1.03	1.05	0.69	1.41	0.7695
CUR	NQ	750	2	752	375	0.602513584	37.47%	4142.88	2.36	476.26	0.56	1.03	0.63	1.43	0.8815
CUR	NQ	375	1	376	375	0.671874584	30.57%	3420.47	1.82	452.86	0.49	0.66	0.37	0.94	0.0195
CUR	NQ	187.5	0.5	188	375	0.81678725	16.17%	2046.33	0.92	390.86	0.34	0.63	0.24	1.03	0.0716
KTO	NQ	3333.33	3.33333	3336.67	1000	0.02749614	92.85%	6540.28	21.02	1242.70	5.72	0.67	0.46	0.88	0.0017
KTO	NQ	1666.67	1.66667	1668.33	1000	0.473291951	43.88%	1708.97	4.48	163.53	0.55	1.35	1.14	1.55	0.0008
KTO	NQ	833.333	0.83333	834.167	1000	0.712427137	17.62%	919.90	2.20	143.91	0.44	1.29	0.97	1.60	0.0767
KTO	NQ	416.667	0.41667	417.083	1000	0.741819983	14.39%	819.91	1.92	140.22	0.43	0.72	0.53	0.92	0.0056
KTO	NQ	2500	5	2505	500	0.055748644	90.79%	5735.20	18.07	990.59	4.47	0.71	0.51	0.91	0.0047
KTO	NQ	1250	2.5	1252.5	500	0.534511945	41.02%	1615.94	4.20	160.14	0.54	1.37	1.16	1.58	0.0006
KTO	NQ	625	1.25	626.25	500	0.781144497	15.38%	851.21	2.01	141.48	0.43	1.36	1.00	1.71	0.0494
KTO	NQ	312.5	0.625	313.125	500	0.768749393	16.67%	891.07	2.12	142.94	0.44	0.65	0.48	0.81	0.0000
KTO	NQ	1666.67	6.66667	1673.33	250	0.17915674	81.31%	3879.46	11.52	488.19	2.08	1.01	0.78	1.24	0.9436
KTO	NQ	833.333	3.33333	836.667	250	0.728640968	25.97%	1165.17	2.88	149.94	0.48	1.87	1.46	2.29	0.0000
KTO	NQ	416.667	1.66667	418.333	250	0.904959456	8.21%	606.50	1.36	128.13	0.38	1.91	1.19	2.64	0.0136
KTO	NQ	208.333	0.83333	209.167	250	0.921931681	6.50%	537.70	1.18	122.54	0.36	1.09	0.64	1.54	0.6888
KTO	NQ	1000	8	1008	125	0.204417993	80.89%	3829.14	11.35	476.55	2.02	0.97	0.71	1.22	0.7942
KTO	NQ	500	4	504	125	0.723330944	31.49%	1325.88	3.35	152.99	0.49	1.57	1.22	1.93	0.0016
